# MUSE Stem Cells Can Be Isolated from Stromal Compartment of Mouse Bone Marrow, Adipose Tissue, and Ear Connective Tissue: A Comparative Study of Their In Vitro Properties

**DOI:** 10.3390/cells10040761

**Published:** 2021-03-30

**Authors:** Domenico Aprile, Nicola Alessio, Ibrahim H. Demirsoy, Tiziana Squillaro, Gianfranco Peluso, Giovanni Di Bernardo, Umberto Galderisi

**Affiliations:** 1 Department of Experimental Medicine, Luigi Vanvitelli Campania University, 80138 Naples, Italy; domenico.aprile@unicampania.it (D.A.); nicola.alessio@unicampania.it (N.A.); ibrahimh.demirsoy@unicampania.it (I.H.D.); squillarot@yahoo.it (T.S.); gianni.dibernardo@unicampania.it (G.D.B.); 2 Research Institute on Ecosystems (IRET), CNR, 80131 Naples, Italy; gianfranco.peluso@cnr.it; 3 Center for Biotechnology, Sbarro Institute for Cancer Research and Molecular Medicine, Temple University, Philadelphia, PA 19122, USA; 4 Genome and Stem Cell Center (GENKOK), Erciyes University, 38280 Kayseri, Turkey

**Keywords:** mesenchymal stem cells, differentiation, adipose tissue, fibroblasts, bone marrow

## Abstract

The cells present in the stromal compartment of many tissues are a heterogeneous population containing stem cells, progenitor cells, fibroblasts, and other stromal cells. A SSEA3(+) cell subpopulation isolated from human stromal compartments showed stem cell properties. These cells, known as multilineage-differentiating stress-enduring (MUSE) cells, are capable of resisting stress and possess an excellent ability to repair DNA damage. We isolated MUSE cells from different mouse stromal compartments, such as those present in bone marrow, subcutaneous white adipose tissue, and ear connective tissue. These cells showed overlapping in vitro biological properties. The mouse MUSE cells were positive for stemness markers such as SOX2, OCT3/4, and NANOG. They also expressed TERT, the catalytic telomerase subunit. The mouse MUSE cells showed spontaneous commitment to differentiation in meso/ecto/endodermal derivatives. The demonstration that multilineage stem cells can be isolated from an animal model, such as the mouse, could offer a valid alternative to the use of other stem cells for disease studies and envisage of cellular therapies.

## 1. Introduction

Mesenchymal stromal cells (MSCs), a heterogeneous population found in stromal tissues throughout the body, contain stem cells, progenitor cells, fibroblasts, and other stromal cells. MSCs are involved in the homeostasis of the body and perform important functions through the secretion of bioactive molecules [1].

In 2010, Dezawa’s research group [2] identified a subpopulation of SSEA3(+) multilineage stem cells within MSCs isolated from human bone marrow. These cells, known as multilineage-differentiating stress-enduring (MUSE) cells, are endogenous cells capable of resisting stress and possess an excellent ability to repair DNA damage [3]. 

The identification of MUSE cells has been demonstrated both in vitro and in vivo, confirming that these cells are an endogenous tissue population. In humans, MUSE cells have been isolated from MSCs of bone marrow and subcutaneous fat, where they represent 2–3% of the population, and from skin fibroblasts where they constitute 1% of the population [4,5].

MUSE cells are self-renewable, not tumorigenic, and can home within damaged tissues. In this context, it has been hypothesized that they may contribute to the daily repair of tissues and organs through spontaneous differentiation in tissue constituent cells [6,7].

Due to the abovementioned properties, MUSE cells are considered to be suitable for regenerative medicine; in fact, it has been proven that they are capable of differentiating into neural cells and facilitate reconstruction after lacunar infarction in immunodeficient mice [8]. MUSE cells also provided restorative effects and robust functional recovery in a rabbit acute myocardial infarction model [9].

There are several findings showing the existence of multilineage stem cell subtypes within MSCs, but studies confirming their existence are controversial [10,11]. In this scenario, the isolation of such stem cells in several mammals may confirm that stromal tissues host multilineage stem cells.

Characterization of MUSE cells isolated from animal tissues is still missing. Recently, only mouse adipose tissue-derived MUSE cells have been extracted and identified for their neuronal differentiation potential in vitro [12], but a thorough analysis of their biological properties has never been performed, and the presence of these cells in different stromal districts has not been verified.

In this study, we isolated SSEA3(+) cells from three different mouse stromal districts —bone marrow, subcutaneous fat, and fibroblasts—and analyzed their biological properties to ascertain if they fulfill the MUSE stem cell phenotype.

## 2. Materials and Methods

### 2.1. Animals

Five C57BL/6 inbred male mice of 3 weeks of age were purchased from Charles River (Wilmington, MA, USA). The animals were handled in compliance with the protocols approved by the Animal Care and Use Committee of University of Campania Luigi Vanvitelli and approved by the Italian Ministry of Health (number 1036/2020-PR).

The mice were euthanized by cervical dislocation, and tissue samples were harvested for the experiments indicated below.

### 2.2. Mouse MSCs and Fibroblasts’ Isolation

We harvested MSCs from the bone marrow of the femurs and tibias of mice by inserting a 21-gauge needle into the shaft of the bone and flushing it with D-MEM (Microgem, Napoli, Italy). The cells from one animal were plated onto two 100-mm dishes with D-MEM low glucose (Microgem) containing 15% ES-FBS (Euroclone, Pero, Italy). After 48 h, we discarded the nonadherent cells and washed the adherent ones with PBS 1X (Microgem). We then incubated the cells for 7 to 10 days in a proliferating medium in order to reach confluence (P0).

We collected MSCs from 300 mg of subcutaneous white adipose tissue surrounding the hips of animals and fibroblasts from the ear connective tissue of the animals. Tissues were digested in a D-MEM solution containing collagenase type II (1 mg/mL) (Sigma, St. Louis, MI, USA) for 30 min at 37 °C. Samples were filtered on cell strainers (70 μm mesh), centrifuged, and washed three times with PBS 1X (Microgem). Cells were plated into 100-mm dishes with D-MEM low glucose (Microgem) containing 15% ES-FBS (Euroclone). We then incubated the cells for 7 to 10 days in a proliferating medium in order to reach confluence (P0). The cells were later trypsinized and expanded until the 3rd in vitro passage (P3).

### 2.3. Harvest of Mouse Tissues and Organs

We harvested leg muscle tissue, liver, and brain. The tissue and organs were collected in cryostat embedding medium (Bioptica, Milano, Italy) or in RNAlater (Qiagen, Hilden, Germany) and stored at −80 °C until immunocytochemistry analysis or mRNA expression analysis.

### 2.4. Culture of MUSE Cells

Confluent MSCs and fibroblasts were collected by 0.25% trypsin-EDTA (Sigma) and were subjected to cell sorting to isolate MUSE cells as described previously [13,14]. In brief, cells were suspended in FACS buffer, which contained 0.5% bovine serum albumin (BSA) (Sigma) and 2 mM EDTA-2H2O (Sigma) in FluoroBrite DMEM (ThermoFisher, Waltham, MA, USA), and were incubated with the anti-mouse SSEA-3 antibody (IBL International, Hamburg, Germany) for 1 h on ice. Cells were then washed three times with FACS buffer and centrifuged at 400× *g* for 5 min. Afterwards, cells were incubated with a secondary antibody, anti-rabbit IgM-FITC (ImmunoReagents, Raleigh, NC, USA) for 1 h on ice, and subsequently washed three times again. Magnetic-activated cell sorting (MACS) was used to collect SSEA-3(+) cells and SSEA-3(−) non-MUSE cells according to the manufacturer’s instructions (Miltenyi, Bergisch Gladbach, Germany). Subsequently, cells were incubated with anti-FITC microbeads (Miltenyi) for 15 min in ice and later washed with FACS buffer. Cells were then loaded on LS columns for magnetic separation.

The collected SSEA-3 positive cells represented MUSE cells and were cultivated in suspension on poly-2-hydroxyethyl methacrylate- (pHEMA) (Sigma) coated petri dishes in a DMEM low glucose medium (Microgem) containing 10% ES-FBS (Euroclone), 4 mM L-glutamine (Sigma, St. Louis, MO, USA), 100 U/mL penicillin-streptomycin (HiMedia, Düsseldorf, Germany), and 2.6% MethoCult (STEMCELL Technologies, Vancouver, BC, Canada) at 37 °C and 5% CO_2_ for 10 days. At the end, the cells were plated for successive experiments or subjected to analysis.

The collected SSEA-3 negative cells were cultured in adhesion in a DMEM low glucose medium (Microgem) containing 10% ES-FBS (Euroclone), 4mM L-glutamine (Sigma), and 100 U/mL penicillin-streptomycin (HiMedia) at 37 °C and 5% CO_2_ for 10 days.

### 2.5. Flow Cytometry Analysis

The MUSE cell populations were washed with PBS and incubated with either anti-CD105 PE-conjugated (Elabscience, Houston, TX, USA), anti-CD90 PE-conjugated (Elabscience), or anti-CD44 PE-conjugated (Elabscience). The antibodies were used according to the manufacturer’s procedures. After 30 min of incubation in the dark with the antibodies at room temperature, cells were washed with PBS 1X and resuspended in FACS buffer for data acquisition on a Guava easyCyte flow cytometer (Merck Millipore, Burlington, MA, USA). We performed data analysis with a standard procedure using easyCyte software. A minimum of 5000 cells per sample were analyzed and gated for forward scatter (FSC) versus side scatter (SSC) channel signals.

### 2.6. Cell Cycle Analysis

For each analysis, 5 × 10^4^ cells were collected and dissociated with yellow tips into single cells. The cells were washed with PBS 1X and were subsequently fixed in 70% ethanol overnight at −20 °C. The samples were washed with PBS 1X and finally were dissolved in a hypotonic buffer containing 100 μg/mL of RNAse A (Promega, Madison, WI, USA) and 40 μg/mL of propidium iodide (Sigma), and incubated for 30 min at RT in dark. The samples were acquired on a Guava easyCyte flow cytometer (Merck Millipore) and analyzed through a standard procedure using easyCyte software.

### 2.7. Cell Proliferation Assay

Cell proliferation was determined by Cell Counting Kit-8 (CCK-8) colorimetric assay (Dojindo Molecular Technologies, Rockville, MD, USA). We seeded 500 cells in 96-wells and CCK-8 reagents were added. Viability was detected by a microplate reader at 450 nm at 6 h (Day 0) and at 1, 2, 3, and 4 days after incubation. The data were expressed as the ratio between the measurement at day “*n*” to the measurement at day “*n* − 1”.

### 2.8. Apoptosis Detection by Annexin V Assay

Apoptosis was detected using a fluorescein-conjugated Annexin V Kit (Dojindo Molecular Technologies) on a Guava easyCyte flow cytometer (Merck Millipore) following the manufacturer’s instructions. In brief, 5 × 10^4^ cells from the several experimental groups were collected from culture dishes and stained with Annexin V-FITC solution containing 7-AAD.

The kit uses two separate dyes (Annexin V and 7-AAD) to identify a broad spectrum of apoptotic and non-apoptotic cells. Annexin V (green) binds to phosphatidylserine on the external membrane of apoptotic cells, while 7-AAD (red) permeates and stains the DNA of late-stage apoptotic and dead cells. Staining allows the identification of four cell populations: non-apoptotic cells (Annexin V– and 7-AAD–), early apoptotic cells (Annexin V+ and 7-AAD–), late apoptotic or dead cells (Annexin V+ and 7-AAD+), and necrotic cells (Annexin V- and 7-AAD+). In our experimental conditions, early and late apoptotic cells were grouped together.

### 2.9. Senescence Detection by Acid Beta-Galactosidase Assay

MUSE cell clusters were collected and dissociated with tips into single cells, then centrifuged and fixed using a solution of 2% formaldehyde and 0.2% glutaraldehyde for 5 min at RT. After that, the cells were incubated with a staining solution containing 1 mg/mL of X-Gal (GoldBio, St. Louis, MI, USA) at 37 °C overnight. The percentage of senescent cells was calculated by the number of blue, b-galactosidase-positive cells out of at least 500 cells in different microscope fields, as already reported [15].

### 2.10. Spontaneous Commitment to Differentiation of MUSE Cells

The spontaneous differentiation capacity of MUSE cells was evaluated according to published protocols [14]. In brief, MUSE cells from cultures in suspension were collected and dissociated mechanically into single cells. Cells were plated into 6-well or 24-well culture dishes that were coated with 0.1% gelatin (Sigma) and grown for two weeks in α-MEM (Microgem) containing 10% ES-FBS (Euroclone), 4 mM L-glutamine (Sigma), and 100 U/mL penicillin-streptomycin (HiMedia). At the end of the two weeks, the cells were fixed for immunocytochemistry (ICC) or collected for RNA extraction.

### 2.11. Immunocytochemistry (ICC) and Immunohistochemistry (IHC)

We grew cells on cover slides and then fixed them in 4% formaldehyde solution for 15 min at room temperature. These samples were used for ICC. The organs and tissues collected in the cryostat embedding medium were cryosectioned to obtain 5 μm cross-sections that were then used for IHC analysis.

We used the following primary antibodies: OCT3/4 (Elabscience), SOX2 (Elabscience), NANOG (Cell Signaling, Danvers, MA, USA), and SSEA3 (IBL International) for stemness evaluation, and desmin (Elabscience), NEFL (Elabscience), and CK-7 (Elabscience) for lineage marker evaluation. All of the antibodies were used according to the manufacturer’s instructions. The secondary antibodies (FITC or TRITC conjugated) were obtained from ImmunoReagents. Nuclear staining was performed by a DAPI mounting medium (ABCAM, Cambridge, UK), and micrographs were taken under a fluorescence microscope (Leica, Wetzlar, Germany). The percentage of positive cells was calculated by counting at least 500 cells in different microscope fields.

### 2.12. RT-qPCR

Total RNA was extracted from cell cultures and tissues using an RNeasy Mini Kit (Qiagen) following the manufacturer’s instructions. We used sequences of mRNAs from the Nucleotide Data Bank (National Center for Biotechnology Information) to design primer pairs for real-time RT-PCR reactions (Primer Express v. 3.0, Applied Biosystems, Foster City, CA, USA); primer sequences are available upon request. We used appropriate regions of GAPDH cDNA as controls and ran real-time PCR assays on an Opticon 4 machine (BIORAD, Hercules, CA, USA). We carried out reactions according to the manufacturer’s instructions using an SYBR green PCR master mix (ABM, Richmond, BC, Canada) and used the 2^−ΔΔ*C*T^ method as a relative quantification strategy for quantitative real-time PCR data analysis.

### 2.13. Statistical Analysis

All experiments were performed in triplicate. Statistical significance was determined using one-way ANOVA and post-hoc tests by JASP v. 0.14.1, which is an open source statistics software supported by the University of Amsterdam (https://jasp-stats.org).

## 3. Results

### 3.1. MUSE Cells Were Successfully Isolated from Mouse Stromal Tissues

We isolated MSCs from bone marrow and subcutaneous adipose tissue of mice. We also isolated fibroblasts from stromal component of mice ears. Isolated cells were cultured in proliferating media for three in vitro passages (3P). Approximately 6 × 10^4^ cells were collected and used to isolate the SSEA3(+) cell population by MACS sorting. The percentage of SSEA3(+) cells ranged between 3.0% and 2.1% of total cell population (Table 1). The isolation of MUSE cells can be performed with cell sorting by flow cytometer of MACS sorting [12,14]. We used the latter one to obtain a higher number of cells.

We designated the isolated SSEA3(+) cells as putative MUSE cells and named them bmMSC-MUSE, aMSC-MUSE, and eFIB-MUSE to indicate cells obtained from bone marrow, adipose tissue, and ear fibroblasts, respectively. The MUSE cells were cultured in suspension for 10 days in vitro (DIV). At that time, we observed numerous discrete clusters of floating cells (spheres) and then performed molecular and biological assays.

**Figure 1:**
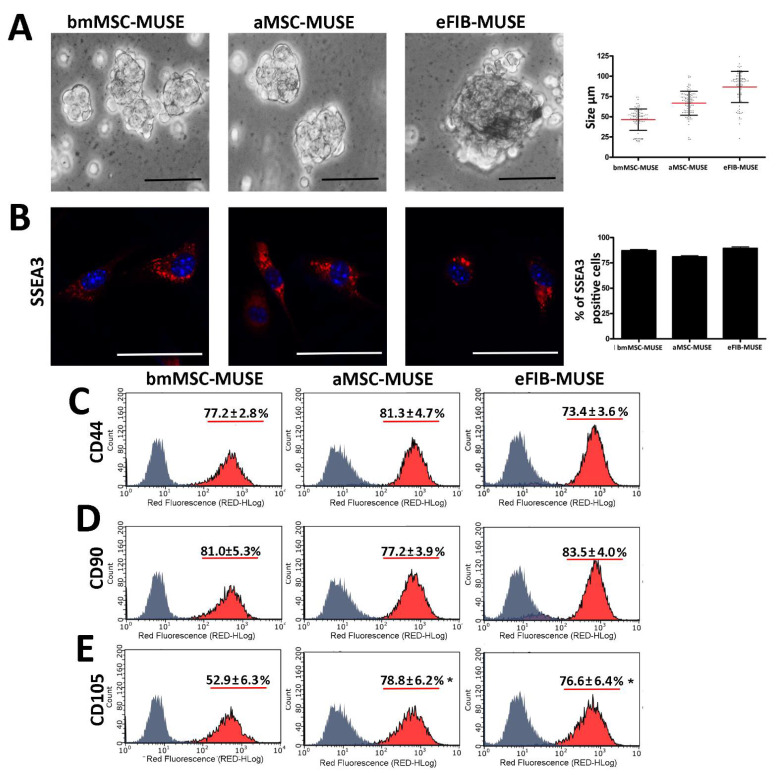
MUSE cells were isolated from bone marrow MSCs (bmMSC-MUSE), subcutaneous adipose tissue MSCs (aMSC-MUSE), and ears fibroblast (eFIB-MUSE). (**A**) Microscopy pictures of MUSE cell clusters showing different sizes. The picture shows representative samples. Scale bars = 50 µm. The graph displays the size of MUSE cell clusters. Each dot corresponds to one MUSE cell cluster. The red bar represents the mean size value in the different cell populations. (**B**) Representative microscopic field of SSEA3 staining (red) in MUSE cells. The nuclei were counterstained with DAPI (blue). The histogram shows the percentage of SSEA3-positive cells in each population. The data are expressed with standard deviation (*n* = 5). (**C**–**E**) Expression of CD44, CD90, and CD105 surface markers measured by flow cytometry in MUSE cells derived from mouse stromal tissues. The data are expressed with standard deviation (*n* = 5). We compared the three MUSE cell populations. The statistical differences among bmMSC-MUSE and the other MUSE cell populations are indicated with * (* *p* < 0.05).

The size and number of clusters generated after 10 DIV were different among the three cell groups, although we seeded the same number of cells (25 × 10^3^/mL) on the poly-hema-coated plates (Table 1). The bmMSC-MUSE had a small cluster average size (48 μm) but were more numerous. The eFIB-MUSE cultures showed a reduction in the number of clusters but had a larger average size (98 μm). Finally, the aMSC-MUSE had an intermediate size (67 μm) (Table 1). The number of cells per cluster was in line with the size. On average, the number of cells per cluster was 15.6 for bmMSC-MUSE, 19.4 for aMSC-MUSE, and 28.3 for eFIB-MUSE (Table 1, Figure 1A). The differences in total number of cells after 10 DIV were not statistically significant among the three cell types (Table 1).

We evaluated whether cell culture conditions allowed the self-renewal of freshly isolated SSEA3(+) cells by determining the percentage of SSEA3(+) cells after 10 DIV. To this end, at 10 DIV, MUSE cells grown in suspension were plated on cover slides for 6 h and immediately analyzed to detect SSEA3 marker by ICC. The three different cell populations (bmMSC-MUSE, aMSC-MUSE, and eFIB-MUSE) exhibited a percentage of SSEA3(+) cells greater than 83% (Figure 1B). There are findings reporting that MUSE cells, besides the expression of the SSEA3 marker, can be positive for some MSC markers, such as CD44, CD105, and CD90 [16]. We evaluated the expression of these markers on the 10 DIV cultures. The three cell populations showed a high expression of CD44 and CD90 surface markers with no statistical differences among them (Figure 1C,D). Differently, the expression of the CD105 marker was more heterogeneous. The aMSC-MUSE and eFIB-MUSE exhibited CD105 expression similar to the other markers, while the bmMSC-MUSE presented a lower CD105 expression as detected by flow cytometry (Figure 1E).

### 3.2. The MUSE Cells from Different Tissues Showed Similar In Vitro Biological Properties

**Figure 2:**
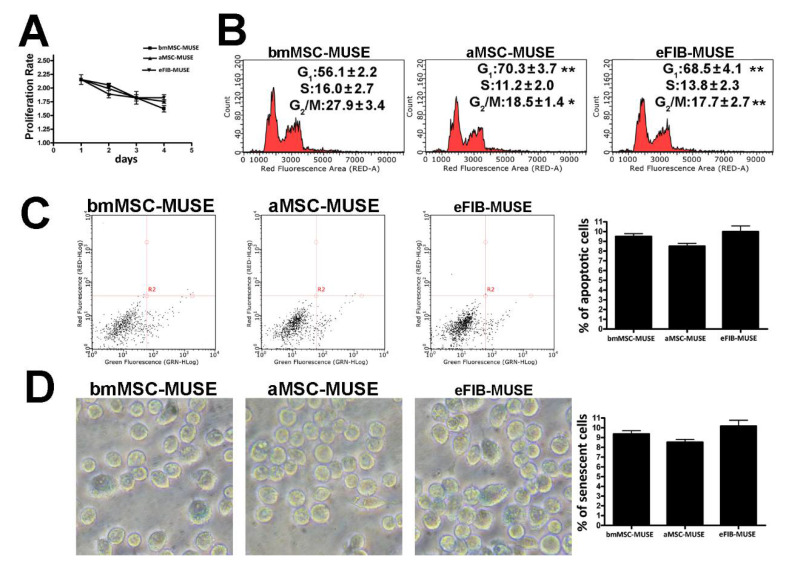
MUSE cells isolated from different stromal tissues showed similar biological properties. (**A**) MUSE cell proliferation was evaluated by Cell Counting Kit-8 (CCK-8) colorimetric assay. (**B**) Representative cell cycle analysis of the MUSE cells isolated from different tissues. The percentages of different cell populations (G1, S, and G2/M) are indicated. (**C**) Representative analysis of MUSE cells apoptosis. The assay identifies early (Annexin V + and 7ADD −) and late (Annexin V + and 7ADD +) apoptosis. Apoptosis is a continuous process, and we calculated the percentage of apoptosis as the sum of early and late apoptotic cells. The histogram shows the mean percentage of Annexin V-positive cells. (**D**) The pictures show representative microscopic fields of senescence-associated beta-galactosidase-positive cells (blue) in MUSE cells cultures. The histograms show the percentage of senescent cells. All experimental data are represented as mean ± standard deviation (SD) of five independent replicates (*n* = 5). We compared the three MUSE cells populations. The statistical differences among bmMSC-MUSE and the other MUSE cell populations are indicated with * (* *p* < 0.05, ** *p* < 0.01).

**Figure 3:**
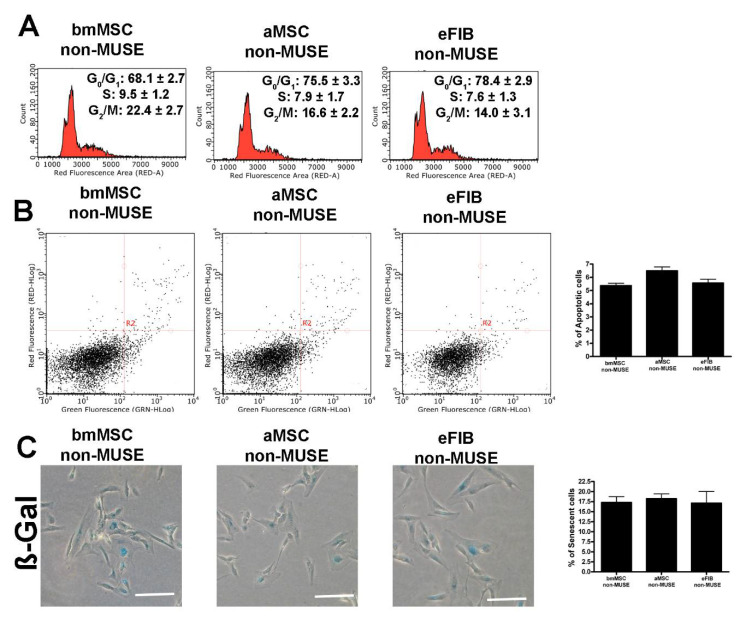
(**A**) Representative cell cycle analysis of the non-MUSE cells isolated from different tissues. The percentages of different cell populations (G1, S, and G2/M) are indicated. (**B**) Representative analysis of non-MUSE cells’ apoptosis. The assay identifies early (Annexin V + and 7ADD −) and late (Annexin V + and 7ADD +) apoptosis. Apoptosis is a continuous process, and we calculated the percentage of apoptosis as the sum of early and late apoptotic cells. The histogram shows the mean percentage of Annexin V-positive cells. (**C**) The pictures show representative microscopic fields of senescence-associated beta-galactosidase-positive cells (blue) in non-MUSE cell cultures. The histograms show the percentage of senescent cells. All experimental data are represented as mean ± standard deviation (SD) of five independent replicates (*n* = 5). We compared the three non-MUSE cells’ populations.

We evaluated the in vitro proliferation potential of bmMSC-MUSE, aMSC-MUSE, and eFIB-MUSE cells. The CCK-8 proliferation assay did not evidence significant differences among the three groups. The observed reduction in proliferation rate between the beginning and the end of CCK-8 assay is related to the increased crowding conditions of cell cultures (Figure 2A). In contrast, the cell cycle analysis revealed a higher percentage of S-phase and G2-phase cells for the bmMSC-MUSE compared to the aMSC-MUSE and eFIB-MUSE; the latter two groups had a percentage of cells in the different phases of the cell cycle very similar to each other (Figure 2B, Supplementary File S1). The non-MUSE cells showed a lower percentage of S-phase cells compared with the corresponding MUSE samples (Figure 3).

The onset of senescence and apoptosis in stem cells could promote deleterious effects in the cell population, such as decreased differentiation potential and impairment in migratory and homing ability [17]. It is of fundamental importance to establish the functionality of stem cells by evaluating cell apoptosis and senescence phenomena in in vitro cell culture. Cellular apoptosis was determined by Annexin V assay and cellular senescence by acid beta-galactosidase assay. These two parameters did not show significant differences among the three cell populations (Figure 2C,D, Supplementary File S1). The senescence levels in non-MUSE cells were higher than in MUSE cells, while the percentage of apoptosis was higher in MUSE cells compared with corresponding non-MUSE samples (Figure 3). The in vitro cultivation always produces cell stress, since it is not a physiological status. Some cells respond to stress by a preferential trigger of senescence; others by promoting cell death. Differences among MUSE and non-MUSE cells may reside in different ways as these populations cope with the stress.

### 3.3. MUSE Cells Expressed Stem Cell Markers

**Figure 4:**
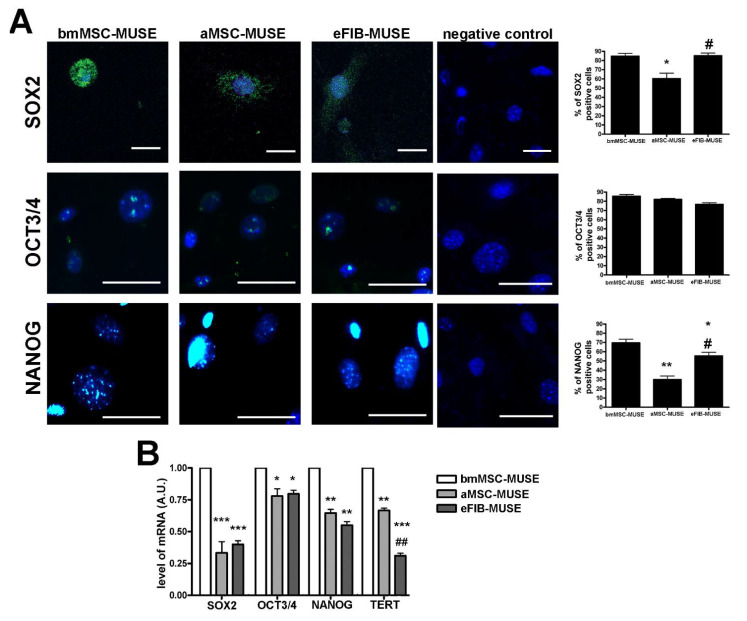
MUSE cells expressed stem cell markers. (**A**) The pictures are representative images of immunocytochemistry for stemness markers: SOX2, OCT3/4, and NANOG, performed on bmMSC-MUSE, aMSC-MUSE and eFIB-MUSE. We performed ICC only with secondary antibodies, as reaction negative control. The nuclei were counterstained with DAPI (blue). The histograms show the percentage of SOX2, OCT3/4, and NANOG-positive cells. The data are expressed with standard deviation (*n* = 5). In the last column is reported the immunostaining performed only with secondary antibody (negative control). The bar indicates 50 μm. (**B**) mRNA expression levels of stem cell genes. The histograms show the quantitative RT-PCR analysis of a group of genes involved in stemness: SOX2, OCT3/4, NANOG and TERT. The mRNA levels were normalized to GAPDH mRNA expression, which was selected as an internal control. Histograms show expression levels in the different MUSE cell and non-MUSE cell populations. Data are expressed as fold changes with standard error (*n* = 5). For each gene, the expression level of bmMSC-MUSE is set as the baseline (the arbitrary value is 1). All experimental data are represented as mean ± standard deviation (SD) of five independent replicates (*n* = 5). We compared the three MUSE cell populations. The statistical differences among bmMSC-MUSE (first column) and the other MUSE cell population are indicated with * (* *p* < 0.05, ** *p* < 0.01, *** *p* < 0.001). The statistical differences among aMSC-MUSE (second column) and the other MUSE cell populations are indicated with # (# *p* < 0.05, ## *p* < 0.01).

**Figure 5:**
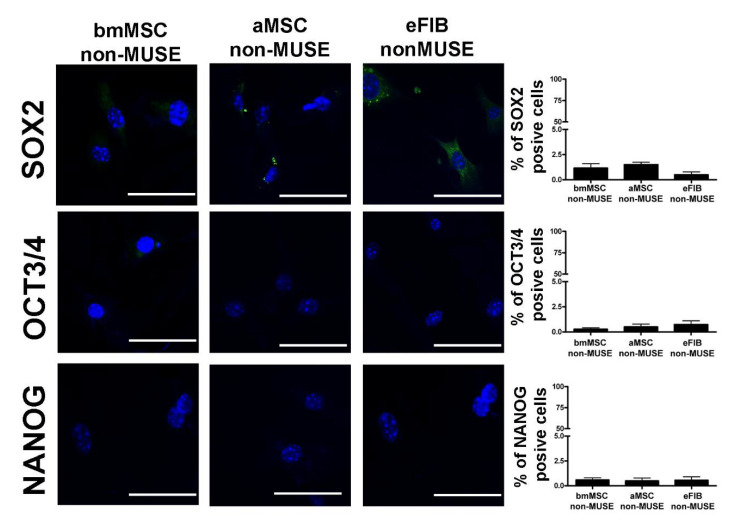
Stem cell markers in non-MUSE cells. The pictures are representative images of immunocytochemistry for stemness markers: SOX2, OCT3/4, and NANOG, performed on bmMSC-non-MUSE, aMSC-non-MUSE and eFIB-non-MUSE. The nuclei were counterstained with DAPI (blue). The histograms show the percentage of SOX2, OCT3/4, and NANOG-positive cells. The data are expressed with standard deviation (*n* = 5). Scale bar = 50 µm.

Like other stem cells, MUSE cells must present stemness properties, i.e., capacity for self-renewal and maintaining of an undifferentiated state as indicated by the expression of stem cell markers. Stemness was assessed in MUSE cells by evaluating the expression of specific stem cell markers: SOX2, OCT3/4, and NANOG [18]. The ICC analysis was performed on MUSE cells at 10 DIV 6 h post-plating on cover slides. The bmMSC-MUSE and eFIB-MUSE cells expressed SOX2 and OCT3/4 markers in the great majority of cells (>75%), while NANOG was expressed in only half of the cells in eFIB-MUSE cultures and in almost 70% of cells in bmMSC-MUSE cultures (Figure 4A). Differently, in the aMSC-MUSE cultures, the great majority of cells expressed OCT3/4, and a lower percentage were positive for SOX2 or NANOG (Figure 4A). The non-MUSE cells isolated from the three analyzed stromal compartments did not express NANOG and OCT3/4 stemness marker, while a small percentage of bmMSC-non-MUSE cells and aMSC-non-MUSE cells showed SOX2 cytoplasmic stain (Figure 5). This data is in agreement with previous findings showing that SOX2 is present in the nuclei of MUSE cells growing in suspension and in the cytoplasm of non-MUSE cells, grown in adherent conditions [19].

We also evaluated the mRNA expression level of stemness genes involved by RT-qPCR. The results showed a higher expression of SOX2 and NANOG in bmMSC-MUSE cells compared to the other two populations. The expression of OCT3/4 mRNA was slightly lower in aMSC-MUSE and eFIB-MUSE cells compared with bmMSC-MUSE cells (Figure 4B). It should be underlined that some differences we observed among protein and mRNA analysis were due to the fact that ICC evaluated the percentage of cells expressing stemness proteins, while the RT-qPCR showed the expression of mRNAs in total cell fraction. In this context, data cannot be directly compared.

The telomerase activity is high in embryonic stem cells to preserve telomere length and cellular immortality. In the great majority of adult stem cells, regardless of their proliferative capacity, the telomerase activity is low or absent [20]. In this context, we decided to evaluate the mRNA levels of the TERT gene, which express the catalytic telomerase subunit, in MUSE cells. The three populations of MUSE cells expressed the TERT mRNA differently from non-MUSE cells, in which this gene was not expressed (Figure 3B). Additionally, the bmMSC-MUSE cells showed a high level of TERT compared to the other cell populations.

### 3.4. MUSE Cells Showed Spontaneous Commitment to Differentiation in Meso/Ecto/Endodermal Derivatives

**Figure 6:**
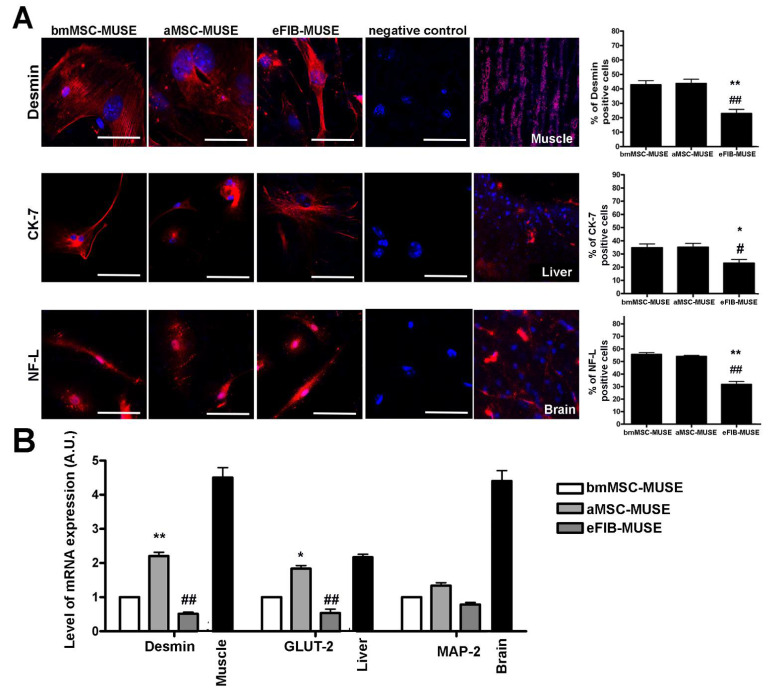
MUSE cells showed spontaneous meso/endo/ectodermal differentiation capacity. (**A**) Representative picture of ICC staining after spontaneous differentiation of MUSE cells. Desmin (red) was used as a mesodermal marker; muscle tissue was used as positive control. CK-7 (red) was used as endodermal marker; liver tissue was used as positive control. NF-L (red) was used as ectodermal marker; brain tissue was used as positive control. We performed ICC only with secondary antibodies, as reaction negative control. The nuclei were counterstained with DAPI (blue). The histograms show the percentage of Desmin, CK-7, and NF-L-positive cells. The data are expressed with standard deviation (*n* = 5). (**B**) mRNA expression levels of meso/endo/ectodermal lineage markers. The histograms show the quantitative RT-PCR analysis of desmin (mesodermal marker), GLUT2 (endodermal marker), and MAP2 (ectodermal marker). The mRNA levels were normalized to GAPDH mRNA expression, which was selected as an internal control. Histograms show expression levels in the different MUSE cell, non-MUSE cell population, and tissues. Muscle, liver, and brain were used as reference tissues for mesodermal, endodermal, and ectodermal markers respectively. For each gene, the expression level of bmMSC-MUSE was set as the baseline (the arbitrary value is 1). All experimental data are represented as mean ± standard deviation (SD) of five independent replicates (*n =* 5). We compared the three MUSE cells populations. The statistical differences among bmMSC-MUSE (first column) and the other MUSE cell populations are indicated with * (* *p* < 0.05, ** *p* < 0.01). The statistical differences among aMSC-MUSE (second column) and the other MUSE cell populations are indicated with # (# *p <* 0.05, ## *p <* 0.01).

**Figure 7:**
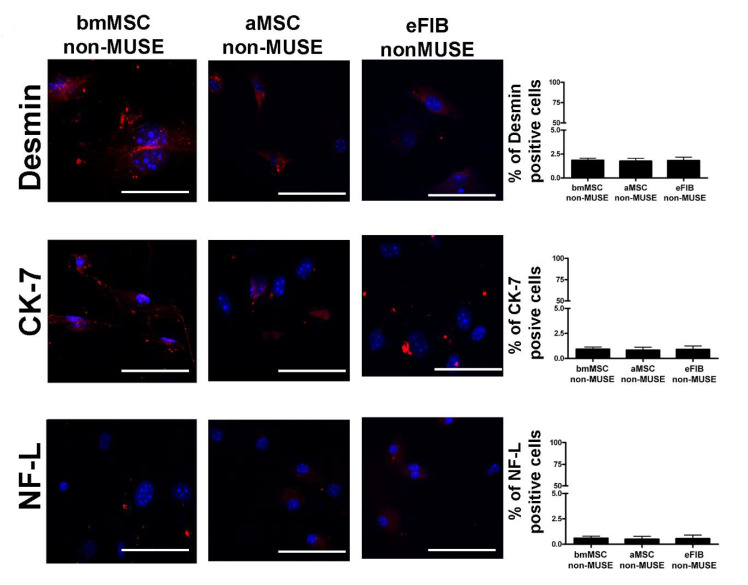
Representative picture of ICC staining after spontaneous differentiation of non-MUSE cells. Desmin (red) was used as a mesodermal marker. CK-7 (red) was used as endodermal marker. NF-L (red) was used as ectodermal marker. The nuclei were counterstained with DAPI (blue). The histograms show the percentage of desmin, CK-7, and NF-L positive cells. The data are expressed with standard deviation (*n* = 5).

Stem cells such as embryonic stem cells (ESCs) and induced pluripotent stem cells (iPSCs) can be grown as cell aggregates called embryoid bodies (EBs). In this state, stem cells spontaneously generate populations of cells expressing genes indicative of lineage commitment in derivatives belonging to three germ layers [21,22]. This phenomenon denotes their multilineage commitment capacity. In order to gain a more comprehensive insight into the MUSE cell capacity to spontaneous lineage commitment, we grew them on gelatin-coated plates for 14 DIV. This experimental condition allows the spontaneous lineage commitment in meso/ecto/endodermal derivatives [14]. The ICC data showed that the three MUSE cell populations expressed desmin, a mesodermal marker, NF-L (neurofilament light), an ectodermal marker, and CK-7 (cytokeratin 7), an endodermal marker (Figure 6A). In non-MUSE cells, the expression was negligible for CK-7 and NF-L. As expected, some non-MUSE cells were desmin-positive, since they are mesodermal derivatives (Figure 7). Of interest, bmMSC-MUSE and aMSC-MUSE cells showed the same percentage of cells committed into the three germ layer derivatives, while eFIB-MUSE cells showed a higher propensity to ectodermal commitment compared to meso/endo lineage specification (Figure 6A). Analysis by RT-qPCR are in line with the immunocytochemical results; in fact, the three cell populations expressed the gene markers of the mesodermal differentiation (desmin), ectodermal differentiation (MAP2), and endodermal differentiation (GLUT2) [14,23] (Figure 6B). The experiments were carried out both on a positive control represented by the reference tissues (muscle, brain, and liver, respectively) and a negative control represented by non-MUSE cells.

## 4. Discussion

MUSE cells are multilineage, endogenous, and non-tumorigenic cells which represent an important promise for future cellular therapies and tissue regeneration therapies. They were initially identified in the human species, but later on, some studies detected them in mice and other mammals such as rabbits and rats [9,24].

In this work, for the first time, an in-depth analysis of the biological properties of mouse MUSE cells isolated from bone marrow, subcutaneous fat, and fibroblasts was performed. Based on the literature available at the moment, this is the first demonstration of the isolation of MUSE cells from mouse ear fibroblasts. These cells represent an abundant source from which MUSE cells could be isolated with a good yield (2–3%). This technique leads to an advance in the study of MUSE cells, as it allows their isolation without euthanizing the animals, thus making it possible to use MUSE cells in cell therapy through the use of an autologous transplant.

The characterization assays showed that after 10 DIV, the vast majority of the cells exhibited the SSEA3 surface marker, while the expression of CD44, CD90, and CD105—which are typical MSC markers—was heterogeneous. This could indicate, as previously highlighted, that there is an intrinsic heterogeneity within stem cell populations [25]. For example, the HSCs, the most-studied type of stem cells, are not a homogeneous population of cells, as previously hypothesized; rather, there is evidence showing that bone marrow hosts at least three subpopulations of HSCs with peculiar differentiation and proliferation features [25]. In this circumstance, it remains to be ascertained why the CD105 marker is heterogeneously expressed within the bmMSC-MUSE population. Indeed, CD105 displays a heterogeneous expression also within the MSCs. In particular, in the mouse MSCs, CD105 exhibits a variable presence affected by passage number and cell confluence and plays a role in differentiation and immunoregulatory properties [26].

The cell cycle analysis of the three populations showed that the bmMSC-MUSE cells had more cells in the S phase than the other MUSE cell populations; cell proliferation, however, was similar in the three groups. Cell proliferation depends on balance between cell division and cell loss (cell death or definitive cell cycle exit). The bmMSC-MUSE cells did not show differences in apoptosis and senescence rate compared to the other two MUSE cell populations, hence it could be hypothesized that bmMSC-MUSE may have a longer cell cycle. There is evidence showing that cell cycle is a major determinant for cell fate decisions, and the idea that the exit from pluripotency is directly coupled to a lengthening of the cell cycle, as previously suggested, has been challenged. In this context, it could be of interest in future research to evaluate the cell cycle length of MUSE cells obtained from different sources [27].

Immunocytochemical analysis demonstrated that a high percentage of MUSE cells expressed stemness genes. OCT3/4 was the most expressed marker in the majority of cells of the three populations. OCT3/4 has been identified as the key factor in the maintenance of stem cells and as the major marker of ESCs [28]. This evidence gives indications on the stemness of the cells that present OCT3/4. We observed a higher variability in the expression of SOX2 and NANOG compared with OCT3/4. This result is in line with studies showing phenotypic cell-to-cell variability within clonal stem cell populations. This variability has two causes. It may derive from gene expression noise or it may reflect stable phenotypic variants [29]. Indeed, MSCs are heterogenous in the content of the earliest progenitor cells: they are composed of progenitors with tri-, bi-, and uni-potential differentiation capability. This occurrence may also be present in MUSE cells and needs further investigation. The spontaneous differentiation process demonstrates the multilineage potential of a stem cell population. In this study, MUSE cells, after culturing on a gelatin-coated plate [14], showed spontaneous commitment to differentiation in meso/ecto/endodermal derivatives.

Globally, our study demonstrated that MUSE stem cells are present in different stromal compartments of mice tissues and, irrespective of their origin, they possess overlapping stemness and multilineage properties.

In future studies, it will be necessary to verify whether the MUSE cells isolated from mouse stromal districts show a homing ability to damaged tissues after intravenous injection or local supplementation, as previously demonstrated for human bmMSC MUSE cells [30], and whether they are capable of differentiating and replacing cells of damaged tissues. Finally, it remains to be assessed whether the MUSE cells isolated from these three sources have different potentials in in vivo studies.

## 5. Conclusions

MUSE cells are multilineage cells that can be successfully isolated from different mouse stromal compartments, such as bone marrow, subcutaneous white adipose tissue, and ear fibroblasts.

The biological properties of the three isolated populations were similar to each other and presented the characteristics that distinguish MUSE cells; however, there were some peculiarities. MUSE isolated from bone marrow cells seemed to have differences in proliferation profiles, in the presence of surface markers and in the gene expression of stem cell markers compared to the other two sources.

The demonstration that multilineage cells can be isolated from an animal model such as the mouse could offer a valid alternative to the use of iPSCs as disease model’s cellular population. MUSE cells exist spontaneously in the body, they do not require genetic/epigenetic manipulations to differentiate in the cell types of interest and, unlike ESCs and iPSCs, they do not present problems related to the formation of teratomas. Finally, the successful isolation of MUSE cells from mouse ear fibroblasts can allow re-implantation studies.

## Figures and Tables

**Table 1:** Isolated cell population characteristics.

	bmMSC-MUSE	aMSC-MUSE	eFIB-MUSE
Yield of SSEA3(+) cells after MACS sorting	3.0%	2.6%	2.1%
Number of cells at 10 DIV/plate	5 × 10^5^ ± 4.1 × 10^4^	5.2 × 10^5^ ± 3.8 × 10^4^	5.4 × 10^5^ ± 4.5 × 10^4^
Number of cluster at 10 DIV/plate	32 ± 4.5	27 ± 4.1	19 ± 3.7
Cluster size at 10 DIV	48 ± 17 μm	67 ± 12 μm	98 ± 15 μm
Cells per cluster at 10 DIV	15.6 ± 5.1	19.4 ± 5.9	28.3 ± 9.8

## Data Availability

Data are reported in the text and in the supplementary file.

## Supplementary Materials

The following are available online at https://www.mdpi.com/article/10.3390/cells10040761/s1, Figure S1: Example of gating strategy for cell cycle analysis performed on MUSE cells and non-MUSE cells.
