# Supplementary material for: MUSE Stem Cells Can Be Isolated from Stromal Compartment of Mouse Bone Marrow, Adipose Tissue, and Ear Connective Tissue: A Comparative Study of Their In Vitro Properties
Source: Cells. 2021 Mar 30;10(4):761. doi: 10.3390/cells10040761 (PMC8065981; doi:10.3390/cells10040761)
Supplement: Supplementary file 1 [file cells-10-00761-s001.pdf]

cells

MDPI

Supplementary file S1

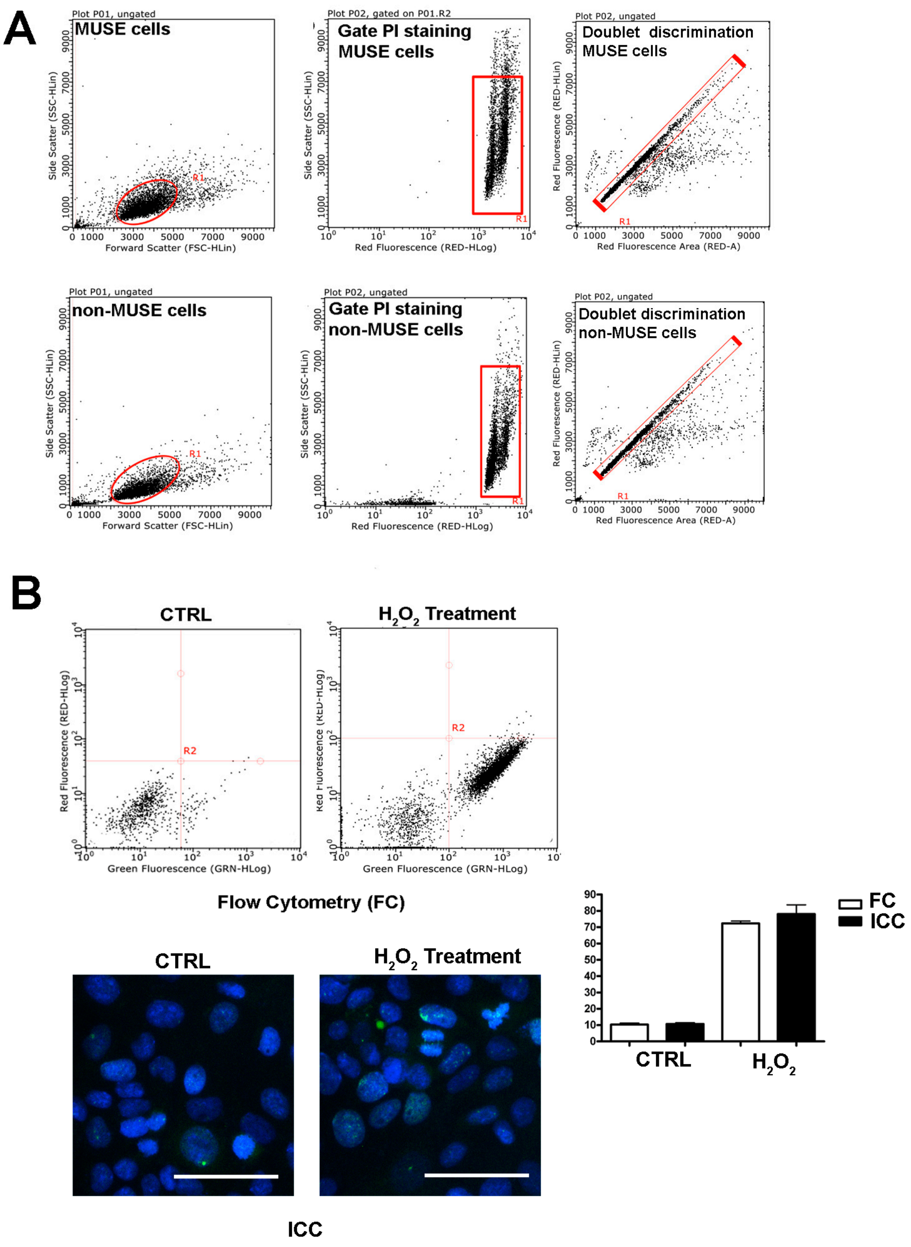

Supplementary file S1 – Panel **A**: Example of gating strategy for cell cycle analysis performed on MUSE cells and non-MUSE cells. An example of doublet discrimination (REDA-REDH) is also shown. The reported gating and doublet discrimination refer to samples obtained from bone marrow.
Panel **B**: Comparison of apoptosis detection with flow cytometry analysis and with in situ annexin V assay.
The detection of apoptosis with flow cytometry was described in materials and methods, below it is reported the *in situ* assay. Bone marrow MUSE cells were treated with 300 µM H_2O_2 for 30 minutes. Following stress, cells were incubated for 24 h in normal culture medium and then apoptosis was evaluated with flow cytometry and *in situ* assay. We used untreated cells as control (CTRL). The figure shows the flow cytometry plots of CTRL and H_2O_2 treated samples. An example of annexin V - positive cells is reported in ICC pictures (Size bar = 50 µm). The histogram shows the percentage of apoptotic cells detected with flow cytometry (FC) and *in situ* assay (ICC). The two methods gave overlapping results.

in situ annexin V assay procedure

Apoptotic cells were detected taking advantage of Alexa 568-conjugated Annexin V (Roche, Italy). To stain apoptotic cells, the culture medium was discarded and cells were incubated with Alexa 568-conjugated Annexin V, diluted 1: 50 in a solution containing 10 mM HEPES, pH 7.4 (Sigma-Aldrich, Italy), 140 mM NaCl (Sigma-Aldrich, Italia), 5 mM CaCl2 (Sigma-Aldrich, Italia), for 15 min at room temperature. Nuclear staining was performed by a DAPI mounting medium (ABCAM, UK), and micrographs were taken under a fluorescence microscope (Leica, Germany). The percentage of positive cells was calculated by counting at least 500 cells in different microscope fields. In every experiment, at least 1,000 cells were counted in different fields to calculate the percentage of dead in culture.
